# Evaluating the Frequency and Characteristics of Unexpected Ovarian Malignancy in Postmenopausal Women Who Have Undergone Laparoscopic Surgery for Adnexal Masses - A Review of Five Years

**DOI:** 10.7759/cureus.42872

**Published:** 2023-08-02

**Authors:** Bhabani Pegu, Thangamuthu Sri Saranya, Sathiya P Subburaj, Rajeswari Murugesan

**Affiliations:** 1 Obstetrics and Gynecology, Jawaharlal Institute of Postgraduate Medical Education and Research, Puducherry, IND; 2 Obstetrics and Gynecology, Jawaharlal Institute of Postgraduate Medical Education and Research, Pondicherry, IND; 3 Biostatistics, Jawaharlal Institute of Postgraduate Medical Education and Research, Puducherry, IND

**Keywords:** tumor marker, unexpected malignancy, laparoscopy, post menopause, adnexal mass

## Abstract

Aim

The aim of this study was to estimate the frequent existence of unexpected ovarian malignant lesions after laparoscopic surgery for an apparent benign adnexal mass and assess its clinical and ultrasound characteristics in postmenopausal women.

Methods

We re-examined the hospital records of 96 cases of postmenopausal women who underwent laparoscopic surgery for benign adnexal mass over five years. The age of the patient, parity, ultrasound findings, tumor markers level, intraoperative findings, and histopathological report were collected. Pearson's Chi-squared test and Fisher's exact test were used for statistical analysis, and a p-value of <0.05 was accepted as statistically significant.

Results

Of a total of 96, benign adnexal mass was in 93 (96.83%), an unexpected ovarian malignancy was observed in two (2.08%) cases, and one (1.04%) had a borderline ovarian tumor. Tumor marker CA-125 was done for all those cases of adnexal mass in postmenopausal women, and not a single case was found to have above 35 IU/ml, defined as the cut-off value for CA-125. Statistically significant differences were observed between the benign and malignant groups in relation to symptoms (p<0.05), ultrasound score (p=0.001), and bilaterality (p=0.013) of the tumor mass.

Conclusion

In postmenopausal women, the critical concern for laparoscopic surgery of benign adnexal mass is unexpected malignancy. So it is essential to select patients carefully for laparoscopic surgery. If a benign-looking adnexal mass turned out to be malignant on the histopathological report, we should try to post the patient for subsequent staging laparotomy as soon as possible.

## Introduction

An adnexal mass (mass of the ovary, fallopian tube, and surrounding connective tissue) is a common gynecological problem. Adnexal masses can be benign or malignant depending upon the nature of the mass, and the diagnosis will occur in 5-10% of women in their lifetime [1]. Clinical presentation of adnexal masses can vary from patient to patient. Depending upon the clinical features and nature of the adnexal mass, management either be a conservative approach or need surgical intervention. The need for surgical intervention is 5.26%, of which 93% of adnexal masses originate from the ovary [2]. As per the Surveillance, Epidemiology, and End Results (SEER) Cancer Statistics Review (CSR) 1975-2014, the lifetime risk of a woman developing ovarian cancer is one in 70 (1.4%), and the risk was slightly reduced to one in 78 (1.3%) in 2018 [3,4]. The risk of malignancy in an adnexal mass increase with age, so postmenopausal women pose a greater risk than the younger age groups. Adnexal mass in postmenopausal women always generates a contradictory situation concerning its diagnosis and management. The use of tumor markers and imaging for screening for ovarian cancer could not rule out malignancy due to inadequate diagnostic sensitivity. Sometimes, performing extensive surgery in the form of staging laparotomy for a benign disease based on a false positive report undue increases morbidity and mortality. On the one hand, there is a lurking fear of missing the diagnosis of malignancy.

Laparoscopic surgery has numerous benefits over open surgery, but a significant limitation of the laparoscopic approach to an adnexal mass is the possibility of encountering an unexpected ovarian malignancy. In addition, laparoscopic surgery does not permit full exploration of the abdominal cavity, and there is a risk of intraoperative rupture of the adnexal mass. This will lead to upstaging of unexpected ovarian cancer and necessitates adjuvant chemotherapy. Further, port-site metastasis is another concern, even in cases of borderline ovarian tumors [5]. A few studies also observed that carbon dioxide in the pneumoperitoneum created during laparoscopy might stimulate the growth of tumor cells [6,7]. The frequency of unexpected ovarian malignancy can be approximately 0.9% even in strictly selected cases, whereas this rate rises to 3% in the case of postmenopausal women [8]. In cases of suspicious adnexal mass in postmenopausal women, the reported frequency of malignancy ranges from 36 to 59% [9,10].

This study aims to estimate the frequency of unexpected ovarian malignancies who underwent laparoscopic surgery for benign adnexal mass and evaluate its clinical and radiological characteristics in postmenopausal women.

## Materials and methods

This is a retrospective study carried out in the Department of Women and Health (WCH) in Jawaharlal Institute of Postgraduate Medical Education and Research (JIPMER), Puducherry. Approval for this study was obtained from the JIPMER Scientific Advisory and Ethical Committee for Human Studies (Approval number: JIP/IEC/2019/175, dated 25/6/2019). A waiver for informed consent was granted because no personal identifying information was collected.

Postmenopausal women underwent laparoscopic surgery for benign adnexal masses from July 2014 to June 2019, as identified from the operation theatre (OT) register. Case records of all these patients were retrieved from the institutional medical record department and reviewed. All the necessary data, including the age of the women, age at menopause, duration of menopause, parity, comorbid factors, presenting symptoms, clinical findings, tumor marker reports, ultrasonography (USG) findings, and computed tomography (CT) findings, were collected in a predesigned proforma. Data were collected on intraoperative findings, procedures performed, complications and need for blood transfusion, and intensive care unit admission. Histopathological reports were retrieved from electronic records. Cases with inadequate data entry in the case sheets and unavailability of the histopathological reports in the system were excluded from the study.

Menopause is accepted to have occurred after 12 months of amenorrhea without apparent pathological cause. Postmenopausal women diagnosed with an adnexal mass on gynecological examination underwent ultrasound. Transabdominal ultrasound (TAS) was performed before transvaginal ultrasound (TVS). Ultrasound findings were categorized as unilocular cysts if there were no septa, solid areas, or papillary projections, and multilocular cysts if they contained septa without solid areas or papillary projections. Ultrasound findings were scored from one to five as per International Ovarian Tumor Analysis (IOTA) Simple Rules, and the risk of malignancy index (RMI) was calculated [11,12]. This simple rule is routinely used for preoperative evaluation to distinguish the type of adnexal masses. The RMI is a mathematical calculation using the mass' USG features, menopausal status, and the numeric value of the CA-125 levels: RMI = U x M x CA125. An RMI cut-off value of 200 was considered an indicator of malignancy.

Ultrasound scans are scored one point for each of the following characteristics: multilocular cyst, bilateral lesions, evidence of solid areas, evidence of metastases, and presence of ascites. M = 1 for premenopausal women and 3 for postmenopausal women, serum CA125 measurement in u/ml.

**Figure 1:**
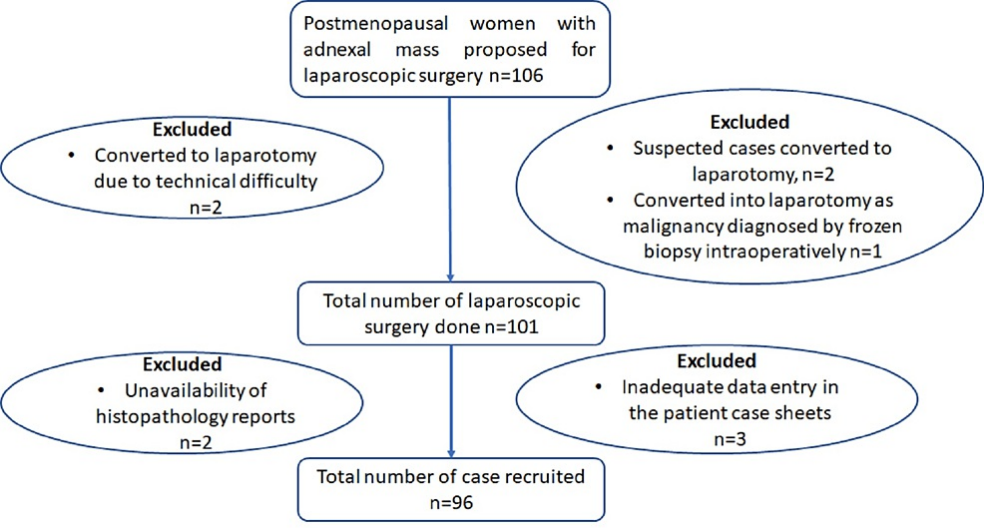
Flow chart on patient recruitment

As per hospital policy, informed consent was taken from the patient before laparoscopic surgery, explaining the procedure to be performed, anticipated complications, and possible need for conversion to laparotomy. At the beginning of surgery, a thorough systematic exploration of the abdomen and pelvis was made. If ascites were present, aspirated or peritoneal wash was given with normal saline, and peritoneal fluid was collected and sent for cytological examination. If malignancy was identified, laparoscopy was converted to laparotomy, and complete tumor staging was performed. Laparoscopically resected ovarian masses were placed into the endoscopic bag. The content of the mass was aspirated inside the bag, and through the primary port, the bag was removed to avoid intra-peritoneal leakage and contamination of the abdominal wall. If mass accidentally ruptured or spillage occurred, thorough irrigation with normal saline (NS) was carried out. Those with suspicious-looking cases for malignancy, patients found to have malignancy on frozen section, and cases converted to laparotomy for some other complications were excluded from the study. Figure 1 shows the recruitment of the patients.

## Results

The study performed laparoscopic surgery for benign adnexal mass in 106 postmenopausal women. Out of 106 cases, five were converted to laparotomy because of dense adhesion, suspicious-looking mass, and malignancy detected in the frozen section, hence excluded from our study. Another five cases were excluded from this study due to inadequate data entry in the case sheets and the unavailability of histopathological reports. So, a total of 96 patients were recruited for this study.

Of the total 96 cases, histopathological reports of 93 patients (96.83%) were benign, one had a borderline tumor (1.04%), and unexpected malignancy was identified in two cases (2.08%). The histological diagnosis of 96 masses operated on laparoscopically is summarized in Table 1.

**Table 1:** Histopathological diagnosis of adnexal mass in postmenopausal women

Benign	N (%)
Dermoid cyst	3 (3.1%)
Endometriotic cyst	2 (2.08)
Serous cyst	36 (37.2%)
Mucinous cysts	29 (30.20%)
Paraovarian cyst	5 (5.2%)
Fibroma	1 (1.04%)
Hydrosalpinx	10 (10.41%)
Simple cyst	7 (7.29%)
Borderline tumor	1 (1.04%)
Malignancy	
Adenocarcinoma	1 (1.04%)
Clear cell carcinoma	1 (1.04%)
Total	96 (100%)

In our study, the mean age of the postmenopausal patients who underwent laparoscopic surgery for adnexal mass was 51.2 years, ranging from 45 to 62 years. Among the study population, 93.75% were multiparous, 5.2% were primiparous, and 1.01% were nulliparous. Out of 86 symptomatic patients, one had malignancy, and another malignancy was observed in an asymptomatic woman. Depending on the size, tumor cases were divided into two groups: tumor size of less than 8cm and more than 8cm. In 83 patients (86.45%), the tumor size was less than 8cm, and the histopathology (HPE) reports of all of these cases were benign except one, which showed a malignant lesion. In the other group with more than 8cm tumor size, malignancy was reported in one case. Bilateral ovarian masses were present in seven patients, out of which malignancy was detected in two cases. Around 11 cases of adnexal masses had multilocular lesions, but most were benign, and only one had malignancy. Though solid components were found in seven adnexal masses, only one of them turned out to be malignant. Ascites was present only in one of the two malignant cases, whereas it was noted in three cases of benign adnexal mass. None of the patients in the study had elevated CA-125 levels. The mean value of CA-125 in benign adnexal mass was 23.59 ± 5.75 and 26.33 ± 4.62 in malignant cases, respectively. One case of borderline tumor was observed, who was asymptomatic and had unilocular but bilateral ovarian tumor without any solid component on ultrasound. Table 2 shows clinical characteristics and ultrasonography features of adnexal mass in postmenopausal women.

**Table 2:** Clinical characteristics of postmenopausal women with adnexal masses (*) Statistically significant

Patients (N=96)	Benign (93)	Malignant (2)	p-value
Age			
Mean ± SD	51.85 ± 4.05	56 ± 4.3	0.155
Range	43 - 62	53 - 61	
Parity			
≤ 2	53	1	>0.05
>2	40	1	
Symptoms			
Symptomatic	85	1	<0.05
Asymptomatic	8	1	
Family history of			
Ca Breast	2	0	-
Ca Ovary	1	0	
Ca Colon	0	0	
USG score			
1	93	1	0.001*
>1	0	1	
USG features			
Bilateral	5	2	0.0133
Multilocular	10	1	0.309
Solid area	6	1	0.205
Ascites	3	1	0.121
Tumor size			
≤8 cm	82	1	0.274
>8 cm	11	1	
CA-125			
Mean ± SD	23.59 ± 5.75	26.33 ± 4.62	0.505
Range	10 - 31	21 - 29	

Statistically significant differences were noticed between benign and malignant adnexal mass in relation to clinical characteristics, the ultrasound scoring (p=0.001), and the bilaterality of the adnexal mass (p=0.013).

All cases of unexpected ovarian malignancy were diagnosed in women over 50 years. Out of two malignant cases, one had a solid component, and it was unilateral but multiloculated without ascites. The second case had ascites, bilateral but unilocular and cystic. Out of two unexpected ovarian malignant cases, one was preoperatively diagnosed as serous cysts, and another was diagnosed as a right-sided dermoid cyst. Two of those cases had adnexal masses with multiple small uterine fibroids. One case with borderline tumor had no ascites, unilocular but bilateral without any solid component. Total laparoscopic hysterectomy with bilateral salpingectomy (TLH with BSO) was done for all those three cases. Table 3 shows the clinical characteristics of patients with unexpected ovarian malignancy and borderline ovarian tumor. All two malignant cases and borderline tumors were called back for a tumor board discussion for further management.

**Table 3:** Details of the patients with unexpected ovarian malignancy and borderline tumors TLH with BSO - total laparoscopic hysterectomy with bilateral salpingectomy

Case	Age	CA-125	Preoperative diagnosis	Tumor characteristics	Surgical procedure	Intraoperative tumor rupture	Histopathology report
1	54	29	Rt dermoid cyst with uterine fibroid	7x5 cm multilocular, solid, no ascites, Intramural fibroid 5x5cm	TLH with BSO	No	Adenocarcinoma
2	53	21	B/L serous cyst with uterine fibroid	8x9 cm, on rt side, 3x4cm on the left side, cystic unilocular, with ascites, multiple small uterine fibroids	TLH with BSO	Yes	Clear cell carcinoma
3.	61	29	B/L serous cyst	9x9 cm on rt side, 3x3cm on the left side, cystic unilocular, with ascites	TLH with BSO	No	Borderline tumor

## Discussion

Minimally invasive surgery has developed as a feasible alternative to laparotomy. Laparoscopic management of adnexal masses is common yet controversial due to conflicting research reports and rapid advances in technology and skill. It is possible to successfully treat adnexal pathology by laparoscopy in selected patients at low risk for cancer. Although most adnexal masses are benign and can be managed by laparoscopy, the challenge is identifying which one would be handled best by laparotomy. There is no specific screening test or preoperative investigation tool to detect ovarian malignancies. Due to an increased incidence of malignant adnexal masses in postmenopausal women, meticulous preoperative investigation has become more essential.

In our study, the frequency of unexpected ovarian malignancy was 2.08%. Whereas a study from Japan, the overall incidence rate of unexpected ovarian malignancy was 1.5%, of which 0.45% was contributed by the postmenopausal group [13]. In a previous study, the author reported the rate of unexpected ovarian malignancy in 0.43% of laparoscopic surgery cases in premenopausal women with benign ovarian mass [14]. The incidence of unexpected malignancy may vary from 11-19% with surgeon's strategies when laparoscopic surgeries were done for adnexal mass that appeared complex on ultrasonography [8,15,16]. However, the rate of unexpected malignancy is estimated to be decreased to 0 - 2.5% if laparoscopic surgeries restrict only those masses that seem to be benign [8,16-18]. Table 4 shows the frequency of unexpected malignancy in adnexal mass reported by previous authors.

**Table 4:** Unexpected malignancy in adnexal masses reported by different authors

Author	Year	Patients (n)	Unexpected malignancy (%)
Canis et al. [16]	1997	230	6.52%
Hidlebaugh et al. [27]	1997	405	1.98%
Malik et al. [28]	1998	292	1.98%
Mettler et al. [29]​​​​​​	2001	493	1.62%
Valentin et al. [30]​​​​​​​​​​​​​​	2006	1066	18.679%
Wahab et al. [15]​​​​​​​​​​​​​​	2011	1161	0.43%
Dermir et al. [31]​​​​​​​​​​​​​​	2012	257	5.84%
Matsushita et al. [14]​​​​​​​​​​​​​​	2014	884	1.5%
Present study	2020	96	2.08%

The unexpected ovarian malignancies are those which were found to be benign on preoperative evaluation. Since malignancy was not suspected even during intraoperative exploration, complete surgical staging of the disease was not done. The diagnosis was made postoperatively when the specimen was examined histopathologically. Out of the three malignant cases, one had an intraoperative rupture, so the disease was upstaged to stage 1C because cyst contents spilled into the abdominal cavity during surgery. However, it is unclear whether tumor cell spillage influences patient prognosis, particularly early-stage ovarian malignancy [24,25]. Operated specimens were removed from the abdominal cavity by the endoscopic bag in the other case. The incidence of intraoperative rupture of the ovarian cyst during adnexal surgery is higher in laparoscopic surgery than in laparotomy [26].

Preoperative evaluation for malignancy is essential in minimizing the risk of unexpected ovarian malignancy. However, it is commonly predictable that ovarian malignancy is difficult to detect during its early stage. Although CA-125 is the most frequently used tumor marker for detecting ovarian malignancy, it can also elevate other gynecological and non-gynecologic conditions [27]. In our study, CA-125 was found to be normal for all the cases of adnexal mass. A study reported that clinical findings, CA-125 levels, pelvic ultrasound, and peritoneal fluid cytological examination could not rule out malignancy due to insufficient diagnostic sensitivity [28]. In the case of postmenopausal women, the risk of malignancy index (RMI) is used as a diagnostic tool for ovarian malignancy, which is recommended by the Royal College of Obstetrics and Gynecology (RCOG) [10]. The International Ovarian Tumor Analysis group developed and validated a logistic regression model (IOTA LR2) [29].

There are a few limitations observed in our study. First, its retrospective design limited our ability to analyze the various risk factors and their effect on adnexal pathology. The second limitation is the small study population. We need to have further research on a large population prospectively. Lastly, we did only a short-term follow-up of those cases with unexpected ovarian malignancy. We did not look for the possibility of cancer dissemination, like port site metastasis and long-term prognosis.

## Conclusions

The possibility of encountering an unsuspected ovarian malignancy at the time of surgery constitutes a significant concern in a benign-looking adnexal mass. A key factor in reducing the likelihood of experiencing an unexpected malignancy when approaching an ovarian cyst by laparoscopy is the appropriate preoperative patient selection for the endoscopic approach. If intraoperative findings are suspicious during laparoscopy, an oncology team should take over the case for a possible immediate staging laparotomy. Suppose ovarian malignancy is not recognized at the time of surgery and is diagnosed only with a final pathological examination; in that case, depending upon the histological type, grade of tumor, and imaging like CT scan, further mode of treatment, either chemotherapy alone or the need of staging laparotomy.
